# Health Tract, No. 189

**Published:** 1865

**Authors:** 


HEALTH TRACT, NO. 189.

### IN THE MIND.

AN old man was shaving himself one day before the fire, but suddenly exclaimed in a great rage to the maid-servant: "I can't shave without a glass! why is it not here?" "Oh!" said the girl, "I have not placed it there for many weeks, as you seemed to get along quite as well without it." The crusty old bachelor (of course he was an old bachelor, or he would not have been so crotchety and crusty) had, for the first time, observed that there was no glass there, and his inability to shave without one, was "in the mind" only, it was imaginary.

A Dutch farmer, who measured a yard through, was one day working in the harvest-field with his little son, and was bitten by a snake. He was horror-struck. When he recovered himself a little, he snatched up his outer clothing, and made tracks for home, at the same time busying himself in putting on his vest; but it wouldn't go on. He looked at his arm, and it seemed to be double its natural size; but tugging at it with greater desperation, he finally got both arms in. But his blood fairly froze in his veins; when he discovered it wouldn't meet by about a foot. By this time he had reached his house, and throwing himself on the bed, exclaimed in an agony of terror: "O mine frow! I'm snake bite! I'm killed! O mine Cot!" But his little bit of a wife, standing a-kimbo in the middle of the floor, burst out into a fit of laughter so uncontrollable, that she was likely to suffocate, and thus beat her husband in dying. The poor man, in his alarm, had endeavored to put on his little boy's vest, and was not swollen at all, except "in the mind."

Many a mother feels fretted and jaded and worn out with the cares of housekeeping, and is almost sick. But at the moment a welcome visitor comes in, full of life and cordiality and cheeriness, in less than five minutes that mother is a different woman; the sky has cleared; the face is lighted up with smiles; and she feels as well as she ever did in her life. Her discouragement, her almost sickness was *not* "in the mind," it was a reality, but the excitement of conversation drove out the wearying blood, which was oppressing the heart, and made it fairly tingle to the finger-points. Mem. Ladies! when you go a visiting, carry smiles and gladness and a joyous nature and a kind heart with you, and you will do more good than a dozen doctors. Most persons have a variety of uncomfortable feelings at times, but they disappear on some exciting occurrence, not because they are merely "in the mind," only imaginary, but because the excited heart wakes up to a new propulsive power, and drives forward the stagnating blood from points where its sluggishness was producing oppression, or actual pain. Mem. 2d. For all, when you are grumpy, bounce up, go ahead, and do something.

